# Radiation therapy improves CAR T cell activity in acute lymphoblastic leukemia

**DOI:** 10.1038/s41419-023-05829-6

**Published:** 2023-05-04

**Authors:** Mayumi Sugita, Takahiro Yamazaki, Mohammad Alhomoud, Jérémie Martinet, Jean-Baptiste Latouche, Encouse Golden, Olivier Boyer, Koen Van Besien, Silvia C. Formenti, Lorenzo Galluzzi, Monica L. Guzman

**Affiliations:** 1 Division of Hematology and Oncology, Department of Medicine, Weill Cornell Medical College, New York, NY USA; 2 Department of Radiation Oncology, Weill Cornell Medical College, New York, NY USA; 3 University of Rouen Normandy, INSERM U1234 Rouen, France; 4 Department of Immunology and Biotherapy, Rouen University Hospital, Rouen, France; 5 Sandra and Edward Meyer Cancer Center, New York, NY USA; 6 Caryl and Israel Englander Institute for Precision Medicine, New York, NY USA; 7 Department of Pharmacology, Weill Cornell Medical College, New York, NY USA

**Keywords:** Acute lymphocytic leukaemia, Immunotherapy, Preclinical research

## Abstract

Autologous T cells engineered to express a chimeric antigen receptor (CAR) specific for CD19 are approved for the treatment of various CD19^+^ hematological malignancies. While CAR T cells induce objective responses in a majority of patients, relapse frequently occurs upon loss of CD19 expression by neoplastic cells. Radiation therapy (RT) has been successfully employed to circumvent the loss of CAR targets in preclinical models of pancreatic cancer. At least in part, this reflects the ability of RT to elicit death receptor (DR) expression by malignant cells, enabling at least some degree of CAR-independent tumor killing. In a human model of CD19^+^ acute lymphoblastic leukemia (ALL), we also observed DR upregulation by RT, both in vitro and in vivo. Moreover, low-dose total body irradiation (LD-TBI) delivered to ALL-bearing mice prior to CAR T cell infusion considerably extended the overall survival benefit afforded by CAR T cells alone. Such an improved therapeutic activity was accompanied by a superior expansion of CAR T cells in vivo. These data encourage the initiation of clinical trials combining LD-TBI with CAR T cells in patients with hematological malignancies.

## Introduction

As of December 2022, no less than six distinct chimeric antigen receptor (CAR) T cell products are licensed by the US Food and Drug Administration (FDA) for the treatment of advanced/refractory B cell neoplasms including acute lymphoblastic leukemia (ALL), B cell lymphoma, follicular lymphoma, mantle cell lymphoma (MCL) and multiple myeloma (MM) [1]. With the exception of idecabtagene vicleucel and ciltacabtagene autoleucel, which recognize the MM-associated antigen TNF receptor superfamily member 17 (TNFRSF17, best known as BCMA) [2], all FDA-approved CAR T cell products target CD19, which is expressed by both malignant and normal B cells [3]. CD19-targeting CAR T cell therapy elicits objective responses (frequently complete remission) in 60-80% of patients with chemotherapy refractory disease, resulting in considerably improved progression-free survival (PFS) and overall survival (OS) in a patient population otherwise difficult to treat [4–7].

While multiple mechanisms of acquired resistance to CAR T cell therapy have been identified, including limited persistence of the CAR T cell product upon infusion and functional CAR T cell exhaustion [8], B cell neoplasms often evade recognition and killing by CAR T cells as they reduce or completely lose expression of the antigenic CAR target, a phenomenon that is commonly known as antigen loss [8]. Considerable progress has been made to extend the persistence and functionality of CAR T cells by genetic alterations of the CAR-coding construct [9]. However, antigen loss remains a major hurdle against optimal CAR T cell activity in patients [10].

Radiation therapy (RT) has been successfully employed to mitigate antigen loss in a preclinical model of pancreatic adenocarcinoma (PDAC) heterogeneously expressing sialyl Lewis-A (sLeA) as a model CAR target [11]. Specifically, sub-cytotoxic RT doses (2 Gy delivered in a single fraction) were shown to promote the expression of death receptors (DRs) including TNF receptor superfamily member 10b (TNFRSF10B, best known as DR5 or TRAIL-R2) by cultured PDAC cells, enabling CAR T cells that produced DR ligands as a consequence of initial CAR signaling to mediate sLeA-independent tumor killing [11]. Moreover, a patient with diffuse large B cell lymphoma who received focal RT (in 5 fractions of 4 Gy each) to skin lesions in a leg (with palliative intent) prior to CD19-targeting CAR T (CART19) cells experienced systemic relapse a few months after CAR T cell therapy (with CD19 negative disease), with the sole exception of the irradiated area [11]. Corroborating the importance of DR signaling in malignant cells for CAR T cell activity, DR defects in the malignant cell compartment have been associated with CAR T cell dysfunction in both preclinical models of ALL and ALL patient samples [12].

Here, we demonstrate the ability of low-dose RT to upregulate DRs including TRAIL-R2 and Fas cell surface death receptor (FAS) in human ALL NALM6 cells, both in vitro and in vivo. However, low-dose total body irradiation (LD-TBI) failed to alter disease course when used as a salvage strategy in NALM6-bearing immunodeficient mice experiencing leukemic relapse likely due to CAR T cell contraction. Conversely, LD-TBI delivered to ALL-bearing mice before CAR T cell infusion considerably extended the OS benefit afforded by CAR T cells alone, despite having no activity on ALL per se. In this setting, LD-TBI maximized the expansion of CART19 cells in the absence of overt, late-phase graft-vs-host disease (GvHD), which instead was observed in tumor-naïve mice receiving LD-TBI followed by CAR T cells [13]. These findings encourage the design of clinical trials assessing LD-TBI prior to CAR T cell infusion in patients with hematological malignancies.

## Materials and methods

### Reagents and cell culture

Human ALL NALM6 cells (ATCC, #CRL-3273) and human lymphoma EL4 cells (ATCC, #TIB-39) were authenticated by STR profiling (University of Arizona Genetics Core or Bio-Synthesis Inc.), and routinely tested for *Mycoplasma spp*. contamination with the PlasmoTest^TM^ Mycoplasma Detection Kit (InvivoGen, #rep-pt1). NALM6 cells were transduced to stably express luciferase and GFP (NALM6-BLIV cells) with the MSCV-GFP-T2A-Luciferase Minicircle (System Biosciences, #BLIV301PA-1) and the calcium phosphate method [14]. All cancer cell lines were cultured in Iscove’s Modified Dulbecco’s Medium (IMDM; Life Technologies, #12440061) supplemented with 10% heat-inactivated fetal bovine serum (FBS; Life Technologies, #10438026) and 100 U/mL penicillin-streptomycin (Life Technologies, #15140122). T cells were cultured in RPMI-1640 medium (Life Technologies, #11875119) supplemented with 10% heat-inactivated FBS.

### Primary samples

Primary leukemia specimens (*n* = 3) were obtained from ALL patients upon ethical approval from Weill Cornell Medicine Institutional Review Board (IRB; # 0909010629, #0910010677). De-identified normal peripheral blood (NPB) samples (*n* = 3) were purchased from the New York Blood Center (New York, NY). Mononuclear cells (MNCs) were isolated by density separation using the Ficoll-Paque separation media (GE Healthcare, #17-1440-03), as manufacturer instructions, and cryo-stored in liquid nitrogen until use.

### Irradiation

Target cells or T cells were diluted at a density of 1-2 million/mL in IMDM or RPMI-1640 medium, respectively, supplemented with 10% FBS and plated in 200-500 µL aliquots into 96- or 48-well plates. After resting 2 h in standard culture conditions, cells were exposed to a single fraction of 0.5-2 Gy irradiation with a Small Animal Radiation Research Platform (SARRP; Xstrahl) or a Faxitron X-ray Cabine (Hologic).

### Flow cytometry—malignant cells

Cells were stained with different cocktails of fluorochrome-conjugated monoclonal antibodies specific for CD3, CD19, CD45, FAS, TRAIL-R1 and TRAIL-R2 (Supplementary Table 1) plus 4′,6-diamidino-2-phenylindole (DAPI; Life Technologies, #D3571), YO-PRO™-1 iodide (Life Technologies, #Y3603), 7-aminoactinomycin D (7-AAD; Life Technologies, #A1310) or Zombie NIR^TM^ (BioLegend, #423105) as viability dyes. Flow cytometry was performed on an LSRFortessa™ Cell Analyzer (BD Bioscience) or a MACSQuant^®^ Analyzer 10 (Miltenyi Biotec). Data were analyzed with FlowJo 10.8.1 (BD Bioscience). Upon exclusion of dead cells, mean fluorescent intensity (MFI) for CD19, FAS, TRAIL-R1, and TRAIL-R2 was assessed. For primary samples, cells were gated on CD45^dim^ blasts (primary ALL samples) or normal CD45^+^CD19^+^CD3^-^ cells. Gating strategies are provided in Supplementary Figs. 1A and 2A.

### Flow cytometry—CAR T cells

Isolated cells from peripheral blood (PB) or bone marrow aspirates (BMAs) were stained with fluorochrome-conjugated antibodies specific for CD45, CD19, CD3, and human truncated epidermal growth factor receptor (EGFRt) (Supplementary Table 1). DAPI, YO-PRO™-1 iodide, or 7-AAD were employed as viability dyes. Acquisition and analysis were performed as detailed above. Percentage of CAR-expressing T cells was evaluated as percentage of EGFRt^+^ cells over CD45^+^CD3^+^CD19^−^ cells.

### Generation of CART19 cells

Second generation CD19-targeting CAR construct consists of an extracellular antigen-binding domain (CD19-scFV), CD8 for hinge and transmembrane domain, 4-1BB co-stimulatory domain, and CD3ζ chain signaling domain followed by EGFRt as a tag. PBMC were collected from buffy coat from healthy donors. T cells were purified on LS columns (Miltenyi Biotec, # 130-042-401) using CD4 and CD8 microbeads (Miltenyi Biotec, #130-045-101 and #130-045-201), were activated with CD3/CD28 beads (T Cell TransAct; Miltenyi Biotec, #130-111-160) and incubated for 24 h. Then, activated T cells were transduced with the lentiviral vector (pCDH-EF1a-CD19 (FMC63)-2nd(4-1BB)-EGFRt; Creative biolabs) carrying the CAR construct. Activated T cells were cultured in TexMACS media (Miltenyi Biotec, #130-097-196) with hIL-7 (155U/mL, Miltenyi Biotec, # 130-095-362) and hIL-15 (290U/mL, Miltenyi Biotec, #130-095-762). CAR positivity was confirmed by expression of EGFRt in CD45^+^CD3^+^CD19^−^ population by flow cytometry. Expanded CART19 cells were frozen and stored in vials in liquid nitrogen before use.

### Quantitative real-time RT-PCR

RNA was extracted with the AllPrep RNA/DNA extraction kit (Qiagen, #80204) and cDNA was synthesized with SuperScript VILO (Life Technologies, #11754250). Real-time RT-PCR was performed on the QuantStudio 12 K Flex Real-Time PCR System (Life Technologies) based on the TaqMan Gene Expression Master mix (Life Technologies, #4369510) and the following TaqMan Gene Expression Assays (Life Technologies): *CD19* (Hs00174333_m1), *FAS* (Hs00236330_m1), *TNFRSF10A* (Hs00269492_m1), *TNFRSF10B* (Hs00366278_m1), *GUSB* (Hs00939627_m1) with FAM/MGB-NFQ or VIC/MGB-NFQ. The following thermal protocol was adopted: UDG decontamination at 50 °C for 2 min, enzyme activation at 95 °C for 10 min, 35–40 cycles of denaturation at 95 °C for 15 s and annealing/extension step at 60 °C for 1 min. Relative abundance of each transcript in experimental versus control conditions was calculated with the 2^-ΔΔCT^ method.

### Cytokine beads assays

Plasma samples were evaluated for the abundance of interleukin 2 (IL2) and interferon gamma (IFNG, best known as IFNγ) with the LEGENDplex™ Human Th Cytokine Panel (BioLegend, #740001), as previously described [15]. Data were analyzed with the LEGENDplex™ Data Analysis Software (BioLegend).

### In vitro cytotoxicity assays

CART19 cells and control T cells (effector cells) were thawed the day of experiment. Target cells (EL4, NALM6) were labeled with cell trace carboxyfluorescein succinimidyl ester (CFSE; life technologies #34554), unless the target cells expressed GFP, and plated into 96-well plates at a concentration of 200,000 cells/mL. Target cells were irradiated at 1 Gy (or 0 Gy for control) and then co-cultured with effector cells at different effector:target (E:T) ratios. Viability of CFSE-stained or GFP^+^ target cells was evaluated by flow cytometry by staining with DAPI. Cytotoxicity was represented as the relative number of live target cells relative to control cells (no effector cells). Control conditions were provided by target cells alone exposed to 0 Gy or 1 Gy, as relevant.

### In vivo experiments

All experiments were approved by Weill Cornell Medicine Institutional Animal Care & Use Committee (IACUC, #2010-0068). NALM6-BLIV cells (1 × 10^6^) were injected i.v. into NOD/SCID *Il2rg*^*-/-*^ (NSG) mice (Jackson Laboratories), followed (10 days later) by engraftment confirmation by flow cytometry on BMAs. On day 11, mice were enrolled to either of the following experiments. In the first experiment, mice (*n* = 3 mice per group) were either left untreated or subject to low-dose total body irradiation (LD-TBI) with a single dose of 0.5 Gy or 1 Gy. In the second experiment, mice (*n* = 24 mice) were allocated to the following treatment cohorts: (1) saline (control, cohort 4; *n* = 3), (2) CART19 cells (5 × 10^6^ i.v.) (cohort 1a; *n* = 3), (3) CART19 cells preceded (4 h) by LD-TBI in a single dose of 1 Gy (cohort 3; *n* = 5); or (4) LD-TBI only (cohort 2; *n* = 3). An additional group of ALL-naïve mice was alongside treated with CART19 cells preceded by LD-TBI (cohort 5; *n* = 5). Finally, a group of ALL-bearing mice treated with CART19 cells was allocated to receive LD-TBI RT at disease relapse (cohort 1b; *n* = 5). LD-TBI was performed by using a RS 2000 Biological Research X-ray Irradiator (Rad Source Technologies). Mice were routinely evaluated for signs of toxicity. Moreover, PB samples and BMAs were routinely obtained to monitor disease progression, CART19 expansion, and CAR persistence on T cells by flow cytometry.

### Statistical analysis

Statistical analysis was performed on Prism v. 9 (GraphPad). The statistical approaches employed are indicated in figure legends. *p* values were considered significant when <0.05.

## Results

### Low-dose RT promotes DR upregulation in ALL cells and sensitizes them to CART19 cells

**Fig. 1:**
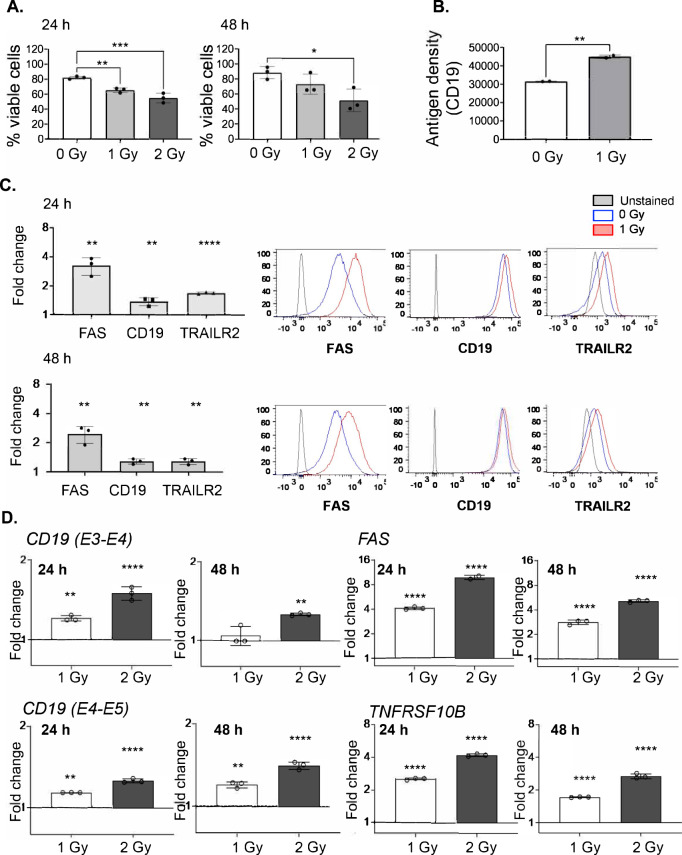
Expression of CD19, FAS, TRAIL-R1 and TRAIL-R2 after low-dose irradiation in vitro. NALM6 cells were cultured in duplicates and irradiated with 0 Gy (control), 1 Gy or 2 Gy. Cells were stained with YoPro-1 to exclude dead/apoptotic cells and expression of CD19, FAS, and TRAIL-R2 in live cells was quantified by measuring mean fluorescent intensity (MFI) for each molecule by flow cytometry. Three or more independent experiments were performed. Fold change of TRAIL-R1 expression and gating strategy were shown in Supplemental Fig. 1. **A** Viability at 24 and 48 h was measured by flow cytometry. Each symbol represents the average of duplicated samples in one experiment and bar represents the mean of 3 independent experiments with the SD. ***p < 0.001, **p < *0.01*, *p < *0.05*, one-way ANOVA. **B** Antigen density of CD19 at 24 h in unirradiated and irradiated cells at 1 Gy was quantified by measuring antigen binding capacity (ABC) using BD Quantibrite^TM^ PE beads by flow cytometry. Representative data is shown from independent replicated experiments. Each symbol represents the average of duplicated samples with the SD. **p < *0.01*, *t*-test. **C** (left) Fold changes of MFI for FAS, CD19 and TRAIL-R2 at 24 (top) and 48 h (bottom) after irradiation at 1 Gy. (Right) Representative histograms of each molecule in live cells irradiated with 0 Gy (blue) and 1 Gy (red) were overlayed with unstained control (gray) at 24 (top) and 48 h (bottom). Each symbol in bar charts represents the average of duplicated samples in one experiment and bar represents the mean of 3 independent experiments with the SD. ****p < 0.0001, **p < *0.01*, *t*-test. **D** Fold changes of transcripts for each target gene relative to control (0 Gy) at 24 and 48 h after irradiation with 1 Gy or 2 Gy were measured by quantitative RT-PCR. The 2^-ΔΔCT^ method was used for analysis. Representative data is shown from independent replicated experiments. Each symbol represents triplicated wells in one experiment with the SD. ****p < 0.0001, **p < *0.01*, one-way ANOVA. CD19 E3-E4 and E4-5 primer sets span exon4-exon5 and exon4-exon5 of CD19 gene respectively.

We set out to interrogate the ability of low-dose RT to alter the expression of surface proteins that are relevant for the activity of CAR T cells on human ALL NALM6 cells engineered to constitutively express GFP and luciferase (NALM6-BLIV cells). To this end, NALM6-BLIV cells were optionally exposed to a single fraction of either 1 Gy or 2 Gy RT in vitro and maintained in culture for 24 or 48 h, followed by the cytofluorometric assessment of cell viability and surface levels of multiple DRs, namely, FAS, TNF receptor superfamily member 10a (TNFRSF10A, best known as DR4 or TRAIL-R1) and TRAIL-R2 by multiparametric flow cytometry (Supplementary Fig. 1A). At these doses, RT was only partially cytotoxic for NALM6-BLIV cells (Fig. 1A). Similarly, the surface abundance of TRAIL-R1 on live NALM6-BLIV cells was only marginally affected by treatment (Supplementary Fig. 1B), Conversely, FAS and TRAIL-R2 levels increased 24 h after RT, an effect that persisted (although with slightly reduced magnitude) 48 h post-RT (Fig. 1B, C). Moreover, we observed an increase in CD19 levels on the surface of irradiated NALM6-BLIV cells (Fig. 1B, C). RT-PCR confirmed that the increase in FAS, TRAIL-R2 and CD19 on the surface of NALM6-BLIV cells 24 and 48 h post-RT is paralleled by an increased abundance in *FAS*, *TNFRSF10B* and *CD19* transcripts (Fig. 1D). Similar findings were obtained in primary samples from ALL patients optionally exposed to a single radiation dose of 1 or 2 Gy ex vivo (Supplementary Fig. 1C).

**Fig. 2:**
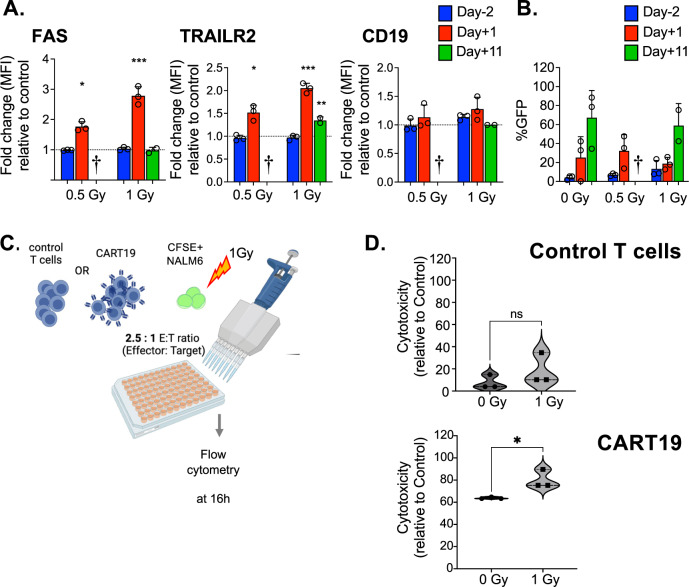
FAS, TRAIL-R2 and CD19 expression in NALM6-BLIV cells in vivo and cytotoxicity by CART19 against target cells in vitro. **A**, **B** NOD scid gamma (NSG) mice were engrafted with NALM6-BLIV cells expressing green fluorescent protein (GFP). After confirming of engraftment, mice were irradiated with 0 Gy (*n* = 3). 0.5 Gy (*n* = 3) or 1 Gy (*n* = 3) on day 13 post tumor injection. Bone marrow aspirates (BMAs) were performed on day -2, day +1 and day +11 post irradiation and FAS, TRAIL-R1, TRAIL-R2 and CD19 expression in NALM6-BLIV cells and tumor burden were evaluated by flow cytometry. Gating strategy is shown in Supplementary Fig. 2. **A** Fold changes of MFI for FAS, TRAIL-R2 and CD19 on day-2 (blue), day+1 (red) and day+11(green) post irradiation for each cohort relative to control (0 Gy). Each symbol represents one mouse and bar represents the mean of each cohort with the SD. ***p < 0.001, **p < *0.01*, *p < *0.05*, one-way ANOVA. **B** Tumor burden (%) in total mononuclear cells (MNCs) was quantified by measuring GFP positivity (%) expressed by NALM6-BLIV cells by flow cytometry. Each symbol represents one mouse and bar represents the mean of each cohort with the SD. No statistical significance in comparison to control (0 Gy), The symbol † indicates that all mice in the cohort were euthanized or died before day+11 due to disease progression. **C** Schematic diagram of cytotoxicity assay by CART19 in combination with or without low-dose irradiation in vitro. NALM6 stained with CellTrace^TM^ CFSE were irradiated with 0 Gy (non-irradiated control) or 1 Gy, and then co-cultured with CART19 or control T cells with effector: target ratio 2.5:1 for 16 h. **D** Cytotoxicity by CART19 or control T cells relative to control was measured by flow cytometry by staining dead cells with DAPI. Representative data from 4 independent experiments is shown. Each symbol represents the average of replicated wells in one experiment and each bar represents the mean of 3 independent experiments. *p < *0.05*, *t*-test.

We next aimed at assessing whether RT can induce DR upregulation on ALL cells in vivo. To this aim, we injected 1 ×10^6^ NALM6-BLIV cells i.v. in NSG mice, enabled the systemic establishment of leukemia for 11 days, and optionally subjected ALL-bearing mice to LD-TBI in a single fraction of either 0.5 Gy or 1 Gy. One or 11 days later, we obtained bone marrow aspirates (BMAs) and assessed CD19 and DR expression on NALM6-BLIV cells by multiparametric flow cytometry (Supplementary Fig. 2A). In line with our in vitro findings (Fig. 1C; Supplementary Fig. 1B), LD-TBI had limited (but statistically significant) effects on TRAIL-R1 expression by NALM6-BLIV cells expanding in vivo (Supplementary Fig. 2B). Conversely, 24 h after LD-TBI with 1 Gy, NALM6-BLIV cells had increased amounts of FAS, TRAIL-R2 and less so CD19 on their surface (Fig. 2A), an effect that persisted 11 days post-TBI only for TRAIL-R2 (Fig. 2A). Moreover, LD-TBI with 0.5 Gy was poorly effective at promoting DR and CD19 upregulation on NALM6-BLIV cells in vivo, the most significant effect being on FAS and TRAIL-R2 24 h post-TBI (Fig. 2A). Consistent with our in vitro cytotoxicity data (Fig. 1A), LD-TBI failed to mediate considerable cytotoxic effects against NALM-BLIV cells expanding in NSG mice, and hence to control in vivo disease progression (Fig. 2B).

We next sought to assess whether sub-cytotoxic RT sensitizes NALM6-BLIV cells to CART19 cell cytotoxicity using in vitro cytotoxicity assays (Fig. 2C). We found that one RT fraction of 1 Gy significantly improves the cytotoxicity of CART19 cells against NALM6-BLIV cells (Fig. 2D). Of note, such an effect (albeit sub-significant) could also be documented with mock-transduced T cells (Fig. 2D). This latter observation reflects at least some degree of alloreactivity, emphasizing the potential of RT as an enhancer of T cell cytotoxicity through both CAR-dependent and independent mechanisms (Fig. 2D).

Taken together, these findings document the ability of sub-cytotoxic RT doses to upregulate DR and CD19 on the surface of ALL cells, in vitro and in vivo, de facto increasing their susceptibility to CART19 cell-mediated killing.

### LD-TBI boosts ALL control by CAR T cells

**Fig. 3:**
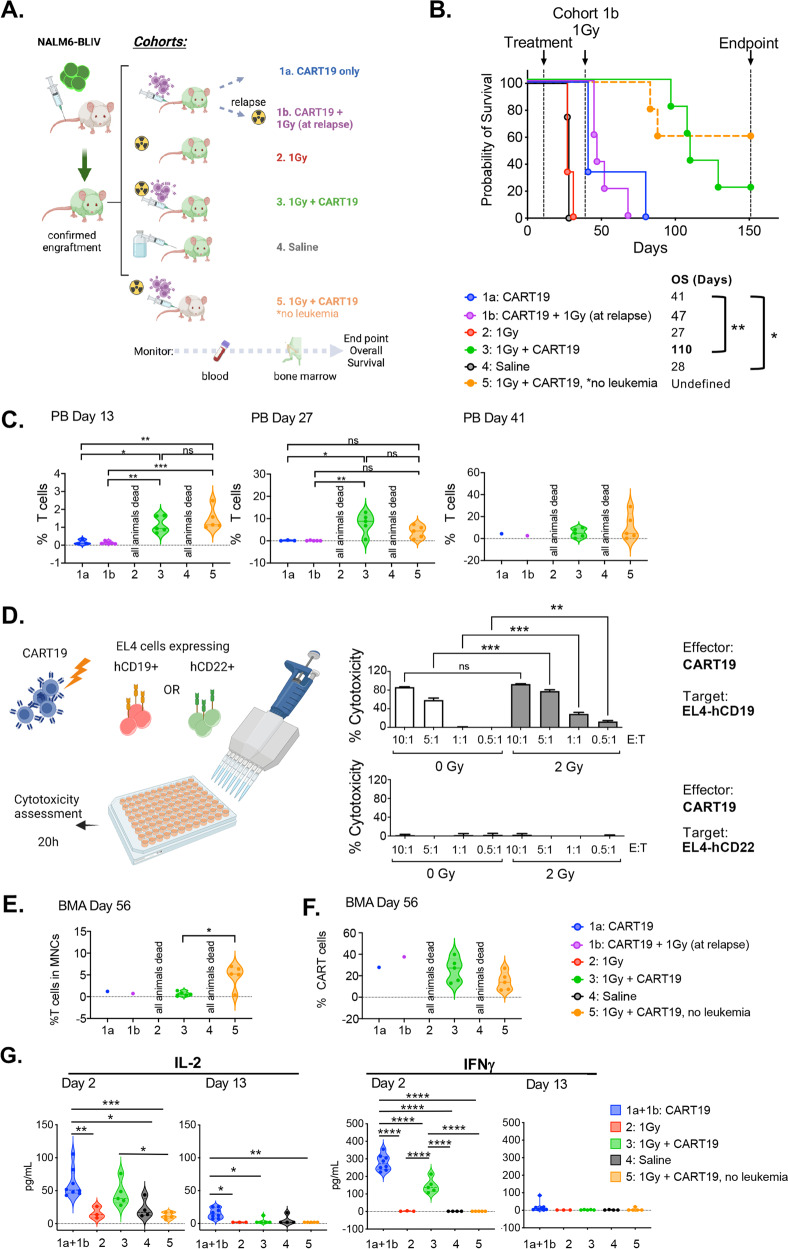
Therapeutic efficacy of CART19 cells combined with LD-TBI against ALL. **A** Schematic diagram of cell line derived xenograft (CDX) cohort treated with CART19 in combination with 1 Gy irradiation. NSG mice were injected with NALM6-BLIV (cohort 1a, 1b, 2, 3, and 4) except cohort 5 (no leukemia). Engraftment was confirmed using cells from bone marrow aspirates (BMAs) by flow cytometry and cohorts were treated as follows; cohort 1a: CART19 only, cohort 1b: CART19 with 1 Gy RT at relapse, cohort 2: 1 Gy irradiation (RT) only, cohort 3: 1 Gy RT and CART19, cohort 4: saline, and cohort 5: 1 Gy RT and CART19. Tumor burden and levels of CART19 in peripheral blood (PB) and BMAs were monitored by flow cytometry and overall survival was assessed. **B** Survival curves of CDX. Cohort 1a (CART19) vs cohort 4 (Saline); *p = *0.0207*, cohort 1a (CART19) vs cohort 3 (RT + CART19); **p = *0.005*, log-rank test. **C** Human T cell (%) in total mononuclear cells (MNCs) in PB on day 13, day 27 and day 41 post treatment. Each symbol represents one mouse and bar represents the mean in each cohort with the SD. ***p < 0.001, **p < *0.01*, *p < *0.05*, ns; no statistical significance, one-way ANOVA. **D** Evaluation of CART19 cytotoxicity after low-dose irradiation. (Left) Schematic diagram of experiment. CART19 were irradiated with 2 Gy and co-cultured with EL4 cells expressing CD19 (target) or CD22 (control) for 20 h under different effector:target (E:T) ratios, 10:1, 5:1, 1:1, or 0.5:1. (Right) Cytotoxicity (%) of non-irradiated CART19 (white bars) or 2 Gy-irradiated CART19 (gray bars) against EL4 expressing CD19 or CD22 are shown. One representative experiment out of two independents experiments is shown. ***p < 0.001, **p < *0.01*, *t*-test. **E** Human T cell (%) in total MNCs in BMAs on day 56 post treatment. Each symbol represents one mouse and bar represents the mean in each cohort with the SD. *p < *0.05*, one-way ANOVA. **F** CAR positivity (%) in human T cells detected in BMAs on day 56 post treatment. Each symbol represents one mouse and bar represents the mean in each cohort with the SD. No statistical significance among cohorts. **G** Interleukin 2 (IL-2) and interferon gamma (IFNGγ, best known as IFNγ) levels in plasma on days 2 and 13 post treatment were measured with LEGENDplex^TM^ by flow cytometry. Cohort 1a and cohort 1b are shown as a combined group (blue, CART19 only), since cohort 1b did not have any other intervention at the evaluated timepoints. Each symbol represents one mouse and bar represents the mean in each cohort. ****p < 0.0001, ***p < 0.001, **p < *0.01*, *p < *0.05*, one-way ANOVA.

We next aimed at evaluating whether LD-TBI could be employed to ameliorate the efficacy of CART19 cells against NALM6-BLIV cells in vivo. To this goal, NSG mice with established NALM6-BLIV ALL were allocated (on day 0) to the following cohorts: (1a) CART19 cells (5 × 10^6^ i.v.), and (1b) CART19 cells plus LD-TBI with a single fraction of 1 Gy on day 27 (upon disease relapse). Additional cohorts included: (2) LD-TBI (1 Gy) on day 0, (3) LD-TBI 4 h before CART19 cells on day 0, (4) saline (control), and (5) tumor-naïve NSG mice receiving LD-TBI shortly before CART19 cells on day 0 (with the intent to evaluate graft-vs-host disease) (Fig. 3A). Mice were routinely monitored for ALL progression by periodic bone marrow aspiration followed by the multiparametric flow cytometry-assisted quantification of GFP^+^ NALM6-BLIV cells (Fig. 3A). Moreover, peripheral blood samples were routinely collected to monitor circulating tumor burden (GFP^+^ NALM6-BLIV cells), T cell abundance and CAR expression on CART19 cells by multiparametric flow cytometry, as well as for the assessment of key circulating cytokines (Fig. 3A).

As expected from our previous findings (Figs. 1A and 2B), LD-TBI delivered to ALL-bearing mice on day 0 had no effect on tumor progression and OS (Fig. 3B). Conversely, CART19 cells significantly controlled ALL progression as they extended the OS of ALL-bearing mice from a median of 28 days to a median of 41 days (Fig. 3B). However, LD-TBI delivered on day 27 failed to ameliorate the therapeutic efficacy of CAR T cells (Fig. 3B). This was likely due to loss of CART19 cells, as RT was delivered upon overt relapse and limited amounts T cells were detected in the peripheral blood of these mice on day 27 (Fig. 3C). Of note, RT did not impair CAR T cell functions, as the in vitro cytotoxic effects of CART19 cells against a CD19^+^ (but not a CD22^+^) target were actually increased (rather than decreased) when CART19 cells were exposed to a single RT fraction of 2 Gy (Fig. 3D).

To our surprise, as early as 13 days after treatment initiation, both ALL-bearing (#3) and tumor naïve (#5) NSG mice receiving LD-TBI plus CAR T cells on day 0 exhibited a considerable expansion of circulating T cells as compared to all other cohorts included in this experiment (Fig. 3C). Such an expansion was sustained as it could still be detected by day 27 and 41 (Fig. 3C). Importantly at the day 56 time point, the BM showed significantly less T cells in ALL-bearing NSG mice successfully controlling ALL upon administration of LD-TBI plus CAR T cells (#3) when compared to tumor-naïve NSG mice receiving the same treatment (#5), suggesting that proliferating T cells may be responsible for GvHD in these animals (Fig. 3E, F). Indeed, ALL-bearing NSG mice receiving LD-TBI plus CAR T cells on day 0 exhibited superior tumor control and OS (median: 110 days) as compared to all other groups (Fig. 3B), in the absence of signs of GvHD. Conversely, 2/5 tumor-naïve mice subjected to the same treatment succumbed to xenogeneic GvHD on day 83 and 88 (Fig. 3B).

Two days after treatment initiation, interferon gamma (IFNG, best known as IFNγ) and interleukin 2 (IL2), which are produced upon CAR T cell activation [16], became detectable above background in ALL-bearing (but not tumor-naïve) mice receiving CAR T cells (but not in mice treated otherwise) (Fig. 3G). Such an early cytokine response could still be documented (although at reduced magnitude) 13 days after treatment initiation (Fig. 3G). Intriguingly, the delivery of LD-TBI prior to CAR T cell administration resulted in decreased IFNγ and IL2 production by CAR T cells at either or both the 2-days and 13-days timepoints (Fig. 3G), potentially linking superior CAR T cell expansion over time to limited early activation of effector functions.

These data suggest that LD-TBI delivered prior to CART19 cells improves their therapeutic activity in the context of superior CAR T cell expansion but absent of GvHD.

## Discussion

Our findings document the ability of low-dose RT to upregulate the expression of FAS and TRAIL-R2 on the surface of human CD19-expressing ALL cells, in vitro and in vivo (Figs. 1, 2). While CD19 loss is likely to contribute to ALL relapse in this model, we were unable to harness DR upregulation by LD-TBI to elicit CD19-independent ALL killing by CAR T cells during leukemic remission as previously done in models of PDAC [11], most likely because CAR T cells were exhausted or in insufficient numbers when we delivered LD-TBI as a salvage approach (Fig. 3). However, we found that LD-TBI delivered shortly before CAR T cell infusion to ALL-bearing NSG mice considerably boosts CAR T expansion in vivo, hence ameliorating ALL control, in the absence of overt GvHD (Fig. 3). Intriguingly, this was paralleled by signs of reduced (not increased) early CAR T cell activation (Fig. 3G), potentially indicating that the global efficacy of CAR T cells may be at least in part inhibited when the very early phase of activation if excessively robust (obviously unless such an early phase is sufficient for disease eradication). That said, the relative contribution of early DR upregulation by ALL cells *versus* improved CAR T cell expansion as driven by LD-TBI on the superior efficacy of this combinatorial regimen remains to be formally assessed.

Our findings emerged from a single ALL model and hence require independent validation in other experimental settings, including immunocompetent mouse models of ALL treated with murine CAR T cells. Moreover, it will be interesting to investigate the potential interaction between RT and CAR T cells in models of hematological tumors other than ALL, such as lymphoma. Despite these and other caveats, our data provide proof-of-concept to harness LD-TBI as a tool to boost the efficacy of CAR T cells in patients with hematological malignancies. Importantly, this therapeutic approach differs from the use of RT as a bridge therapy prior to CAR T cell administration, as in the latter setting RT is employed at therapeutic doses and delivered focally to the tumor [17]. On the contrary, we propose to harness LD-TBI as purely immunomodulatory (rather than cytostatic/cytotoxic) agent [18, 19], an approach that appears at low risk for unexpected toxicities, potentially immediately before CAR T cell administration after conventional focal RT as a bridge therapy. It will be important to assess the therapeutic potential of this approach in clinical trials.

## Supplementary information

Suppl. Material

Checklist

## Data Availability

The data generated in this study are available upon request to the corresponding authors.
