# Supplementary material for: Radiation therapy improves CAR T cell activity in acute lymphoblastic leukemia
Source: Cell Death Dis. 2023 May 4;14(5):305. doi: 10.1038/s41419-023-05829-6 (PMC10160073; doi:10.1038/s41419-023-05829-6)
Supplement: Supplementary file 1 — Suppl. Material [file 41419_2023_5829_MOESM1_ESM.pdf]

Supplemental Figure 1

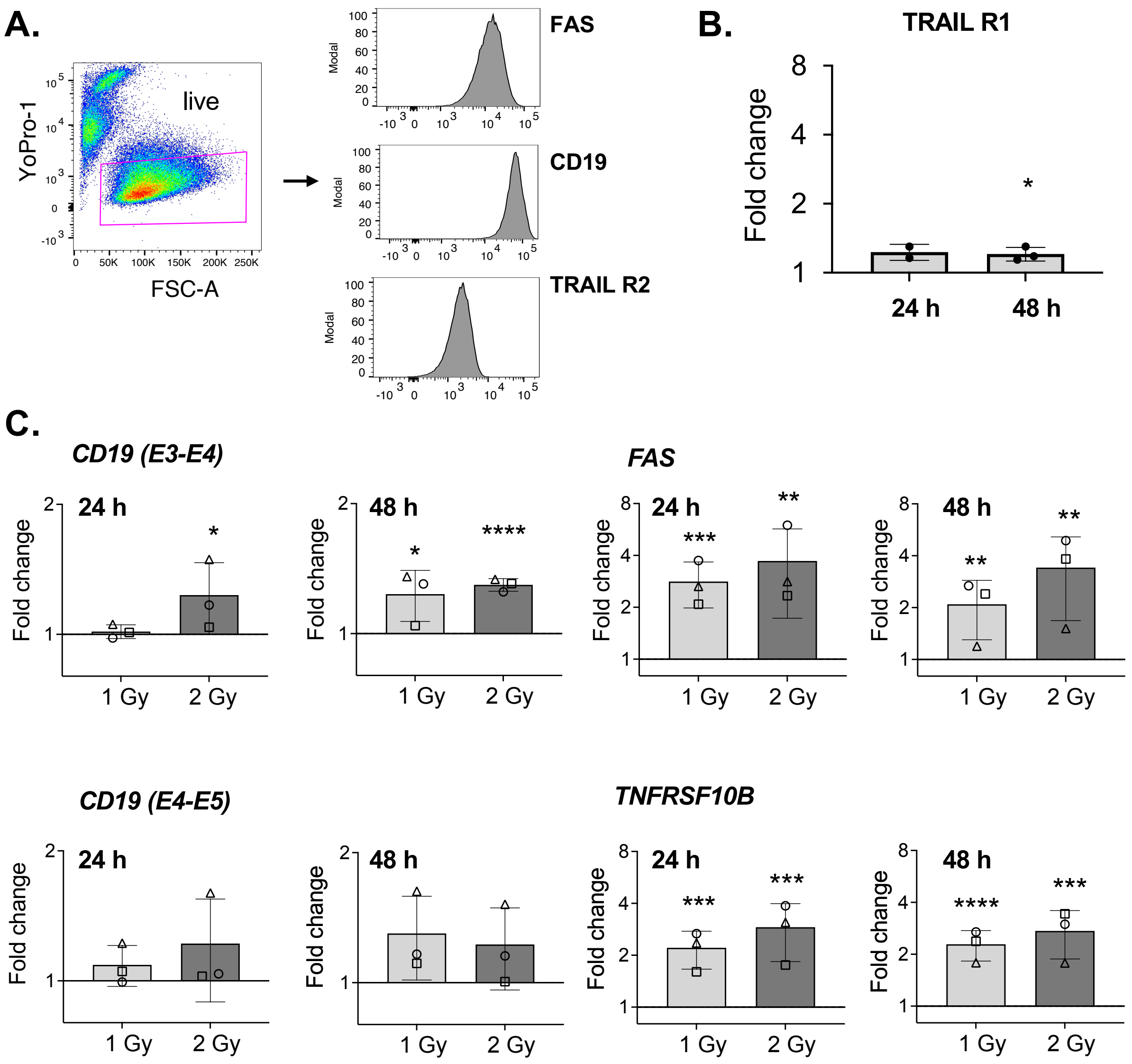

Supplemental Figure 1. **A,** Measurement of CD19, FAS, and TRAIL-R2 expression by flow cytometry. Dead/apoptotic cells were excluded by staining with YoPro-I and mean fluorescent intensity (MFI) was measured for each target molecule in live single cells. **B.** Fold change of MFI for TRAIL-R1 in NALM6 cells 24 and 48 hours after irradiation with 1Gy. Each circle represents the average of duplicated samples in one experiment and bars represent the mean of 3 independent experiments with SD. * p<0.05, t-test. **C.** Fold change in *CD19*, *FAS* and *TNFRSF10B* transcripts relative to control (0 Gy) were quantified 24 and 48 hours after irradiation of primary B lymphoblastic leukemia (B-ALL, n=3) sample with 1 or 2 Gy, by quantitative RT-PCR. The 2(-$\Delta\Delta_{Ct}$) method was used for analysis. Each symbol represents the average of triplicated wells in one sample and bars represent the mean of 3 primary samples with SD. CD19 E3-E4 and E4-5 primer sets span exon4-exon5 and exon4-exon5 of *CD19*, respectively. ****p<0.0001, ***p<0.001, **p<0.01, *p<0.05, two-way ANOVA, Turkey's multiple comparison test.

Supplemental Figure 2

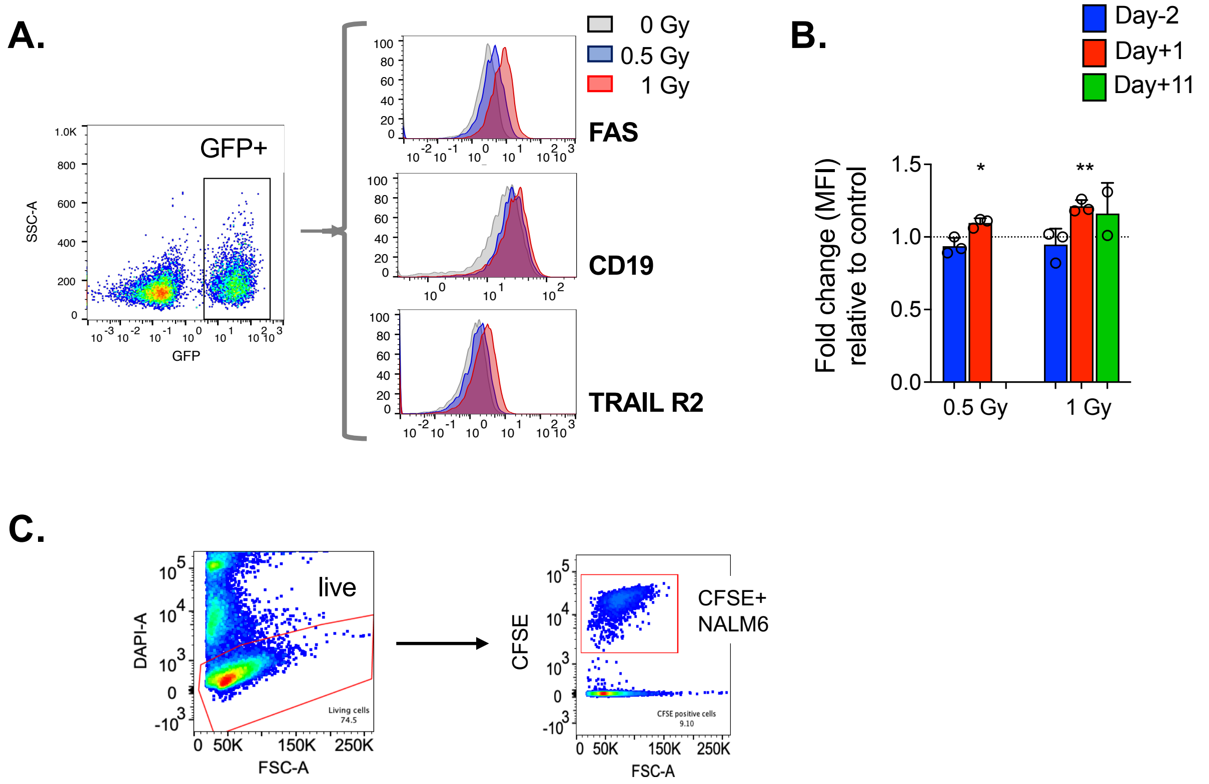

Supplemental Figure 2. **A.** Gating strategy for quantification of CD19, FAS, TRAIL-R1 and TRAIL-R2 expression on NALM6-BLIV cells *ex vivo* by flow cytometry. After gating on single live cells by staining with Zombie NIRTM as a viability dye, GFP^{+} cells were gated on and mean fluorescent intensity (MFI) was measured for each target molecule. Representative overlayed histograms of one mouse from each cohort on day+1 after irradiation at 0.5 Gy (blue) or 1 Gy (red) for each target molecule are shown with 0 Gy control (grey). **B.** Fold MFI change of TRAIL-R1 in NALM6-BLIV cells from the bone marrow on day-2 (blue), day+1 (red) and day+11(green) post irradiation (0.5 Gy or 1 Gy) was quantified relative to control (0 Gy). Each symbol represents one mouse and bars represent the mean of each cohort with SD. **$p<0.01$, *$p<0.05$, one-way ANOVA. **C.** Gating strategy for cytotoxicity assay. Single live cells were gated on by staining with DAPI as a viability dye, and frequency of viable NALM6 cells pre-stained with CFSE was measured by flow cytometry.

Supplementary Table 1. Flow antibodies and reagents

Flow antibodies	Vendor	Cat #
anti-human CD19 PE (HIB19)	e-Biosciences	12-0199-42
anti-human CD19 PerCpCy5.5 (HIB19)	BioLegend	302230
anti-human Fas PECy7 (DX2)	BD Phaingen	561633
anti-human TRAIL-R1 (CD261) BV421 (S35-934)	BD OptBuild	744791
anti-human TRAIL-R2 BV510 (B-529)	BD OptBuild	745057
anti-human CD45 APC-H7 (2D1)	BD Biosciences	560178
anti-human EGFR PE (Hu1)	R&D	FAB9577P
anti-mouse CD45 PE-Cy5 (30-F11)	BioLegend	103110
anti-human CD8 PECy7 (SK1)	BioLegend	344712
anti-human CD3 BV605 (SK7)	BioLegend	344836
anti-human CD3 BV711 (SK7)	BioLegend	344838
anti-human CD4 APC (SK3)	BioLegend	344614
4′,6-diamidino-2-phenylindole (DAPI)	Life Technologies	D3571
7-aminoactinomycin D (7-AAD)	Life Technologies	A1310
YOPRO-1 iodine	Life Technologies	Y3603
Zombie NIR	BioLegend	423105
CellTrace™ CFSE Cell Proliferation Kit	Life Technologies	C34554
